# Spectroscopic Terahertz Imaging at Room Temperature Employing Microbolometer Terahertz Sensors and Its Application to the Study of Carcinoma Tissues

**DOI:** 10.3390/s16040432

**Published:** 2016-03-25

**Authors:** Irmantas Kašalynas, Rimvydas Venckevičius, Linas Minkevičius, Aleksander Sešek, Faustino Wahaia, Vincas Tamošiūnas, Bogdan Voisiat, Dalius Seliuta, Gintaras Valušis, Andrej Švigelj, Janez Trontelj

**Affiliations:** 1 Department of Optoelectronics, Center for Physical Sciences and Technology, Savanoriu Ave. 231, Vilnius 02300, Lithuania; rimvydas.venckevicius@ftmc.lt (R.V.); linas.minkevicius@ftmc.lt (L.M.); vincas.tamosiunas@ftmc.lt (V.T.); bogdan.voisiat@ftmc.lt (B.V.); dalius@pfi.lt (D.S.); gintaras.valusis@ftmc.lt (G.V.); 2 Faculty of Electrical Engineering, University of Ljubljana, Trzaska 25, Ljubljana 1000, Slovenia; aleksander.sesek@fe.uni-lj.si (A.S.); andrej.svigelj@fe.uni-lj.si (A.Š.); janez.trontelj1@guest.arnes.si (J.T.); 3 Instituto de Investigacao e Inovacao em Saudeand, Instituto de Engenharia Biomedica, University of Porto, Rua do Campo Alegre, 823, Porto 4150-180, Portugal; fwahaia@fc.up.pt

**Keywords:** compact THz sensors and components, THz imaging systems, multispectral THz imaging, medical THz imaging

## Abstract

A terahertz (THz) imaging system based on narrow band microbolometer sensors (NBMS) and a novel diffractive lens was developed for spectroscopic microscopy applications. The frequency response characteristics of the THz antenna-coupled NBMS were determined employing Fourier transform spectroscopy. The NBMS was found to be a very sensitive frequency selective sensor which was used to develop a compact all-electronic system for multispectral THz measurements. This system was successfully applied for principal components analysis of optically opaque packed samples. A thin diffractive lens with a numerical aperture of 0.62 was proposed for the reduction of system dimensions. The THz imaging system enhanced with novel optics was used to image for the first time non-neoplastic and neoplastic human colon tissues with close to wavelength-limited spatial resolution at 584 GHz frequency. The results demonstrated the new potential of compact RT THz imaging systems in the fields of spectroscopic analysis of materials and medical diagnostics.

## 1. Introduction

Radiation of terahertz (THz) frequency offers non-destructive and non-ionizing ways of imaging and spectroscopy stimulating the development of THz technologies for security, medicine, biochemical, and materials science [1,2]. Recent achievements in the THz field have triggered new applications in biology and biomedicine with the particular aim of exploring the specificity of fingerprint spectra of materials [3,4]. Development of stand-alone, real-time, and frequency sensitive imaging schemes is of prime interest due to the measurement time, portability, and price issues of currently available THz systems [5]. Recently a compact room temperature (RT) imaging system developed for security needs demonstrated the ability to screen objects inside packages without opening them and to draw a materials map via principal components analysis [6,7]. Most practical applications need real time measurements and a high signal to noise ratio (SNR), therefore, frequency-selective THz sensors with fast response time, high dynamic range, and low noise-equivalent-power (NEP) are required.

Compact, sensitive, and large-format THz cameras delivering images in real time have been developed for many practical applications [8,9,10]. In Reference [8] a THz bolometer camera was able to achieve fast scanning of a large field of view of opaque scenes in a complete body scanner prototype. As an option, the uncooled microbolometer THz focal plane array (FPA) was reported by the company NEC [9]. Values of minimum detectable powers per pixel were comparable with those of other compact THz detectors, such as uncooled field effect transistor (FET) THz sensors and cooled bolometer arrays demonstrating large potential in the frequency range of 0.3–4.3 THz. On the other hand, the French Institut National d’Optique (INO) has developed a THz imaging system capable of detecting concealed weapons or hidden objects behind drywall, and for non-destructive testing military applications [11]. The imaging system was based on the THz cameras built at INO, for example, the THz-optimized IRXCAM-THz-160 and IRXCAM-THz-384 cameras that support the 160 × 120 and 384 × 288 uncooled microbolometer pixel array with a pixel pitch of 52 μm and 35 μm, respectively. The sensitivity of INO’s THz detectors was determined by NEP values reaching up to 25 pW/√Hz and 76 pW/√Hz at frequencies of 4.25 THz and 2.54 THz, respectively [11]. The NEP dependence on the wavelength was explained by differences in pixel size, detector bandwidth, and pixel responsivity. As an alternative, a simple mass producible THz detection array has been developed within standard complementary metal-oxide-semiconductor (CMOS) technology [12]. The sensors based on a thin-film absorber on a membrane and process-integrated thermopiles have provided a 5 ms thermal time constant, together with a wavelength independent NEP of 1 nW/√Hz. Such a THz detection array enabled real-time imaging at 50 frames/s with a signal-to-noise ratio of 10 for an optical intensity of 30 μW/cm^2^. Recently, a significant advance toward compact, low-cost real-time THz imaging systems have been proposed integrating metamaterial absorbers with bolometric vanadium-oxide sensors [13]. The absorber was realised directly in the layers of a standard 0.18 μm CMOS process but the micro-bolometer sensors were defined by post-processing procedures. An absorption magnitude of 57% at 2.5 THz, a minimum NEP of 37 pW/√Hz and a thermal time constant of 68 ms for the sensor were experimentally assessed. Very recently, direct comparison of commercial thermal detector arrays for off-axis THz holography and real time THz imaging have been performed employing a far infrared gas laser system as a powerful THz radiation source [14]. The results revealed that during the same experiments the SNR of the pyroelectric camera was significantly lower in comparison to the bolometeric one at around 3 THz. Moreover, THz cameras have not yet reached the high lateral resolution of the thermal micro-bolometers both in number of pixels and in pixel pitch. Thus, a search for a new physical mechanism for efficient THz radiation detection and the performance optimisation of single THz sensor is necessary.

FET-based THz sensors have been proposed for efficient rectification of THz waves [15,16]. Currently, the most promising device for THz detection at RT seems to be the THz antenna-coupled FETs (TeraFETs) consistently developed for multispectral THz imaging up to 4.25 THz [7,17]. The theory of plasma rectification suggests that the response of the TeraFETs extends into THz region far beyond the cut-off frequency of Si transistor [16,18]. However, in practice optimal TeraFET performance was observed at frequencies of about 600–700 GHz due to electrical losses in parasitic RC components and interconnects [7,18] as well as due to optical losses in the substrate due to antenna effects [19]. The main advantage of Si technology is that the THz devices can be fabricated within a standard CMOS process and supplementary modules such as amplifiers and multiplexers can be integrated on the same chip [10,20].

As an alternative, InGaAs-based bow-tie diodes—thermoelectric-based RT THz sensors—were developed for the frequency range up to 1 THz [21]. The NEP value of such THz sensors was found below 4 nW/√Hz [22] and an attractive possibility to fabricate a monolithic THz detectors array was demonstrated [23]. Furthermore, suitability of the bow-tie diodes for spectroscopic needs was confirmed and compared *versus* a commercial pyro-electric THz sensors by measuring packed samples at discrete fingerprint frequencies in the range of 0.58–2.52 THz [22]. The measured THz absorbance of the samples was found to be in a good agreement with Fourier spectroscopy data allowing the authors to perform principal components analysis of the admixtures. At that time THz imaging system size reduction possibilities were limited by the usage of a bulky THz source—an optically pumped gas laser. Separate group presented a stand-alone, portable system for high resolution real-time THz imaging based on the quantum cascade laser (QCL) emitting at 3.4 THz in continuous-wave mode at a cryogenic temperature of 50 K with an output power of 1 mW [24]. Real time THz imaging capability with a spatial resolution of 2.5 times the wavelength was demonstrated in the system with a commercial uncooled microbolometer camera. A confocal microscopy THz system based on a cryogenically cooled 2.9 THz QCL providing a large contrast enhancement via a lateral and axial resolution better than 70 μm and 400 μm, respectively, was demonstrated [25].

On the other hand, antenna-coupled titanium (Ti) microbolometric sensors have been proposed for fast and sensitive THz detection at RT [26,27]. Our group developed dipole-type THz antennas on a thin silicon-nitride-oxide (SiNO) membrane and used them for efficient THz radiation coupling to the air-bridged microbolometer [28]. A typical narrow band microbolometer THz sensor (NBMS) equipped with a 300 GHz frequency dipole antenna exhibited the response time of 1 µs, sensitivity of 300 V/W, and NEP as low as 14 pW/√Hz. The performance of the NBMS in a vacuum was up to three times better as compared to the operation at room environment [26,28].

In this work, particular attention has been given to the investigation of spectral selectivity of the NBMSs. For this purpose an air-bridged Ti microbolometer was coupled to a double-dipole- or cross-dipole-type antenna. The frequency response was measured in a wide frequency range of 0.1–1.5 THz via the recently proposed quasi-optical THz detectors characterization technique [29]. The NBMS was found to be a very sensitive and frequency selective device. Therefore, a compact multi-frequency THz imaging system based on the NBMSs was developed and applied for inspection of plastic packages and for principal components analysis of explosive simulators.

Over the past few years improvement in the compact RT THz sensors brought very sophisticated THz imaging systems into being [1,5]. However, the total size of the system is still limited by the dimensions of commercially available THz components like mirrors, beam splitters, waveguides, and lenses. The Fresnel zone plates being thinner, lighter and in some cases more effective in comparison with identical diameter and focal length refractive lenses can be used to reduce the system size. Recently, a compact focusing component—the THz zone plate with integrated resonant filter apertures (TZP)–has been developed for 0.76 THz frequency [30,31]. Quite complex setup based on an optically pumped THz laser has been used at that time to proof the concept and to illustrate the operation principles of the TZP. And it was not possible to demonstrate wavelength limited operation without a stable THz source emitting Gaussian mode beam. Nevertheless, such a novel diffractive lens was found to be more efficient in terms of frequency selection and high aspect-ratio focusing in a single device. Moreover, the laser-ablated zone plates can be integrated directly into the bottom surface of the semiconductor substrate on which the THz sensors were fabricated [32]. Integration of THz components into a single device has the advantages of size, price, permanent stable construction and alignment with the THz sensor.

Terahertz science and technology provided new ways for supplementary diagnosis and therapy of the skin, colon, and gastric cancer [33,34,35]. In general, cancer environment causes increased blood supply to affected tissues and an increase of water content [34,36]. This fact acts as a natural contrast mechanism for THz imaging [37]. Moreover, a structural change occurring in the affected tissues was also demonstrated as a contributing factor to the THz contrast [33,35,38].

In this work thin diffractive optics was developed for efficient THz beam focusing at a frequency of 584 GHz. The TZP lens with a 16.5 mm diameter and a 10 mm focal length was fabricated on a 30 µm thick metal foil. The focusing performance was obtained measuring two-dimensional profiles of THz beam along an optical axis by the recently proposed technique [39]. The numerical aperture (NA) for the TZP was of about 0.62 which allowed us to increase the spatial resolution of THz images in comparison to that measured with commercial parabolic mirrors (PMs) roughly by 25%. Finally, the compact THz imaging system enhanced with novel TZP was proposed for biomedical microscopy applications. For this purpose dehydrated human colon tissues were imaged at a frequency of 584 GHz. Higher contrast and close to wavelength limited spatial resolution were observed in the measured THz images comparing non-neoplastic control and neoplastic tumor tissues.

## 2. Antenna Coupled Titanium Microbolometer Sensors

**Figure 1:**
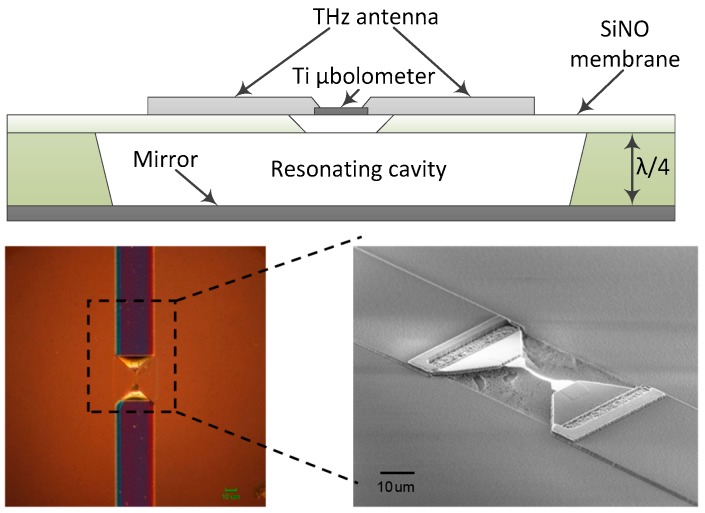
A schematic view of the antenna-coupled Ti-microbolometer sensor (top); microscope image of the fabricated Ti-microbolometer and central part of THz antenna: flat-top view (bottom left) and side-3D view (bottom right). Note a tiny air-bridged Ti wire positioned in the center of the antenna.

Narrow band THz antennas were developed for multispectral THz imaging applications in the frequency range from 0.2 THz to 2 THz. A schematic view of the antenna-coupled microbolometer THz sensor is shown in Figure 1. Selectivity was enhanced by adjustment the dipole antenna geometry and resonant-cavity design, *i.e.*, the back side reflection mirror was positioned at the quarter wavelength distance. An air-bridged Ti-microbolometer and THz antenna were processed on a few microns thin SiNO membrane in order to increase the sensitivity via reduction of thermal losses of the device. A metalized bottom plate under the SiNO membrane acted as a perfect reflector and enhanced the spectral selectivity of the THz sensor [26,28].

**Figure 2:**
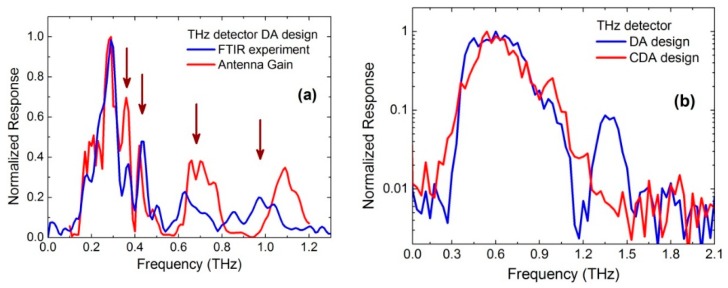
A normalized response spectrum of the THz sensor with a double dipole antenna optimized for operation at a frequency of 300 GHz: the results of experiment and calculation (**a**); Frequency response of the THz sensor with the dipole antenna (DA) and cross-dipole antenna (CDA) design optimized for the 600 GHz frequency (**b**); Note that the amplitude scale is linear (**a**) and logarithmic (**b**), respectively.

The THz sensors were fabricated on a 4′′ size Si substrate. Several types of dipole antennas of various dimensions and complexity were designed The response spectrum of the NBMS was measured with a custom-designed Fourier transform infrared (FTIR) spectrometer in vacuum at RT. The mercury-arc lamp of the FTIR spectrometer served as the THz radiation source [29]. Measured response spectra are shown in Figure 2. As it was expected the NBMS with double dipole antenna (DA) design demonstrated the maximum sensitivity at a specified resonant frequency, namely 300 GHz or 600 GHz. Experimental results were compared with gain calculations of the DA obtained using the ANSYS HFSS computer program. Data comparison is shown in Figure 2a. A reasonably good agreement between modeling and experimental data in the vicinity of the fundamental frequency of the antenna was achieved. Moreover, higher order resonances of the antenna coupled THz sensor were observed in the spectrum as indicated by vertical arrows in Figure 2a. Note that the simulated antenna gain spectrum nicely fitted the experiment data in the whole range up to 1.3 THz frequency.

Measured response spectra of the THz sensor with different THz antenna designs optimized for 600 GHz frequency are shown in Figure 2b. Note that the results are shown in the semi log scale. Although the NBMS with DA design has a quite large side peak at around 1.3 THz, the CDA design provided a single-peak response characteristic without any side peaks.

**Figure 3:**
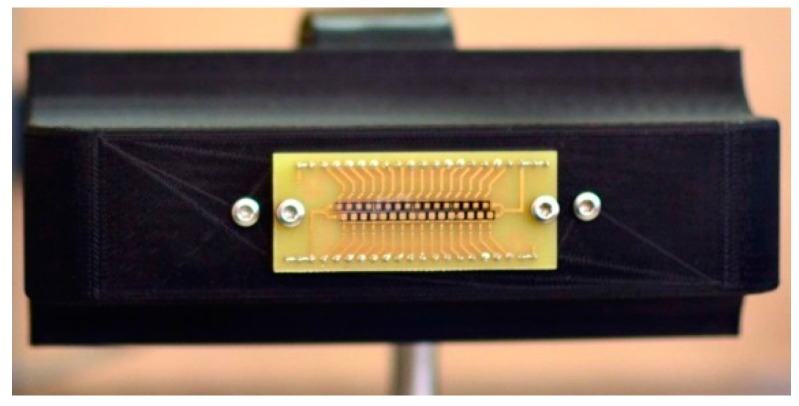
A photo of dual frequency 2 × 16-pixels THz camera, where the top line of pixels is designed for 600 GHz and the bottom line—for 300 GHz. The pixel pitch is 2 mm.

The first dual frequencies linear THz camera was developed for low intensity THz radiation detection at RT. The NBMS camera was successfully applied by the company (Luvitera Ltd., Vilnius, Lithuania) for beam profile monitoring of the pulse-emitters used in the THz time domain spectrometers (TDS) [40]. Figure 3 shows a photo of the linear THz camera of 2 × 16 pixels with each line optimized for 300 GHz and 600 GHz frequencies, respectively. The main advantages of the camera were high sensitivity of 300 V/W and the NEP as low as 14 pW/√Hz. The pixel pitch and the pixel size were 2 mm and 0.6 mm × 0.6 mm, respectively. The relative detectivity D^∗^ of the pixel, which equals the square root of the absorber area divided by the NEP, was estimated to be 4.3 × 10^9^ cm /√Hz/W. These values compare well with the detectivity of other RT THz detectors [12,14] and the NBMS camera was good enough to monitor the beam profile of the photoconductive THz antenna emitting power of 10 µW in real time without any additional optical components [28,40]. Moreover, such a THz camera can be applied for real-time imaging using minimum intensity of the THz optical field of 2 μW/cm^2^ at a 30 Hz refresh rate with a ten to one signal to noise ratio. Although the THz camera acquires images more than ten times faster in comparison to the single pixel raster-scan technique, further the sample pixel by pixel scan method was implemented in order to avoid unsearchable discretization effects [22].

## 3. Multispectral THz Imaging

**Figure 4:**
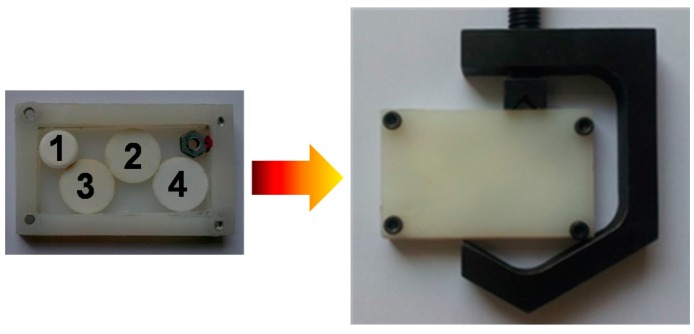
A photo of test pellets packed inside a plastic container. Samples were prepared by mixing PTFE powder with 1—actose, 2%–10% of tartaric acid, 3—Mixture of 5% of tartaric acid and 5% of sucrose. The reference PTFE pellet labeled as 4 and a small hex nut were added for calibration purposes.

**Figure 5:**
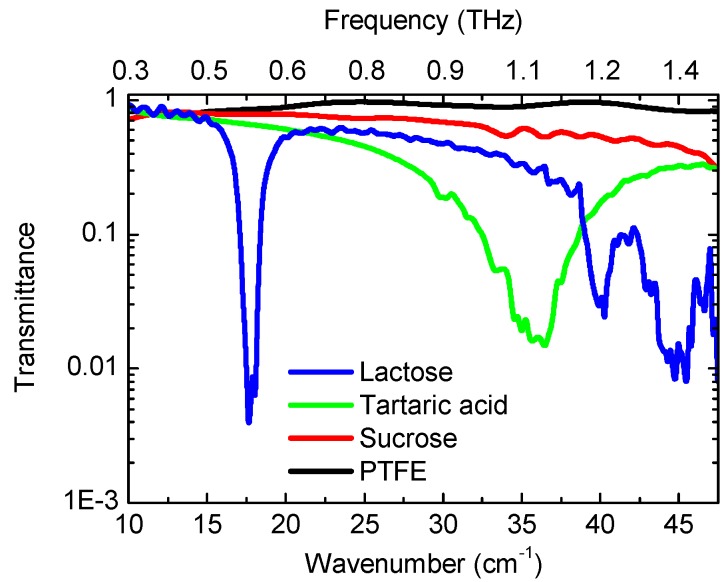
Transmittance spectrum of the samples with lactose, tartaric acid, sucrose, and reference PTFE.

High sensitivity antenna coupled NBMS were employed for the multispectral THz imaging experiment The test samples were prepared as white pellets composed of polytetrafluoroethylene (PTFE) powder and different admixtures. The details on fabrication procedure can be found elsewhere [6,7]. The samples were packed in a plastic container as shown in Figure 4. Transmittance spectra of the samples obtained by FTIR spectroscopy in vacuum are shown in Figure 5. The pellets with the content of lactose and tartaric acid demonstrated different absorption signatures, namely a sharp absorption peak at frequencies of 0.55 THz and 1.1 THz, respectively. Therefore, the NBMS can be applied for spectroscopic THz imaging in a similar manner as it was proposed for THz beam profiling of the photoconductive pulsed-emitters [28,40]. Our developed 300 GHz frequency resonant sensors can serve for reference signal measurement and a 600 GHz frequency sensors covering spectrum range 0.5–0.7 THz can provide the discrimination between different chemical components, such as lactose, tartaric acid, sucrose, *etc.* [6,7].

**Figure 6:**
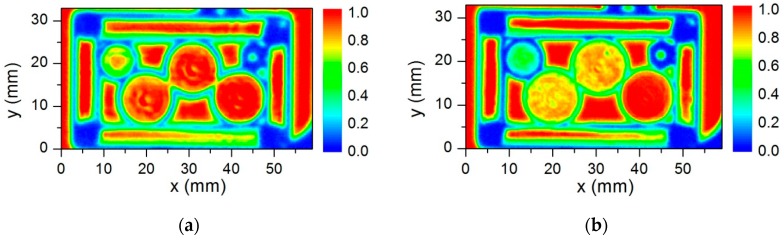
The THz transmission image of plastic container measured with the system based on the NBMS designed for the frequencies of 300 GHz (**a**) and 600 GHz (**b**), respectively.

The samples inside the plastic container were measured at resonant frequencies of the NBMS’s. The results are shown in Figure 6. The pellet containing lactose had higher absorbance both at 300 GHz and 600 GHz frequencies. While other pellets with tartaric acid demonstrated higher absorption only at the frequency of 600 GHz in accordance with the results obtained by FTIR spectroscopy. This abnormal absorbance of lactose pellets seen only with the 300 GHz sensors was attributed to side optical effects such as defocusing and scattering being more pronounced in smaller diameter samples and thicker samples (see Figure 4).

**Figure 7:**
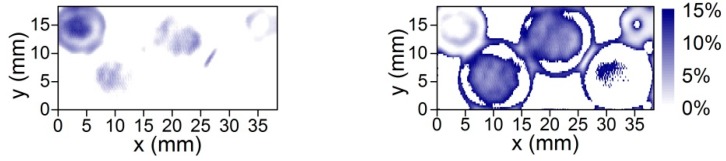
Content distribution map of the lactose (**Left**) and tartaric acid (**Right**) in the pellets packed in a plastic container as shown in Figure 4. The intensity scale is linear and represents the amount of the component across the pellet.

Principal component analysis was performed by using measured THz transmission images. Obtained spatial content distribution of lactose and tartaric acid is shown in Figure 7. The blue color represents the amount of the sample across pellet as described elsewhere [6,7]. Note that admixture maps were obtained without opening the plastic container. In this way the suitability of the antenna-coupled microbolometer sensors was demonstrated for multispectral THz imaging applications. Further research will be oriented towards THz antenna technology development to increase resonant frequencies to 2.5 THz [8,9] and integration of compact THz components into a single device [32].

## 4. Diffractive THz Components for High Spatial Resolution Imaging

**Figure 8:**
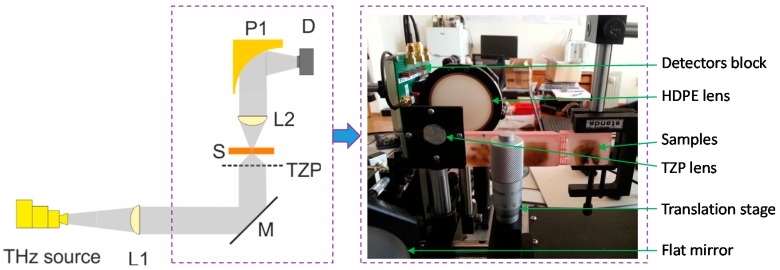
THz imaging setup (**Left**), where M is a flat mirror; L1 and L2—PE lenses, TZP—a diffractive lens [30,31], S—a sample on three-axes translation stage, P1—a parabolic mirror, D—compact THz detectors. The photograph (**Right**) shows the arrangement of the components in the experiment.

The experimental setup of the compact all-electronic RT THz imaging system is shown in Figure 8. The source of THz radiation was an electronic multiplier chain (Virginia Diodes, Inc., Charlottesville, VA, USA) delivering of about 0.8 mW power at a frequency of 584 GHz. Emitted THz radiation was collimated with 12 cm focal length polyethylene (PE) lens L1. The THz beam reflected by 2 inch diameter flat mirror M was focused with the THz lens TZP and directed to sample S. Transmitted THz radiation was collimated with 6 cm focal length PE lens L2 and focused onto THz detector D with PM (P1) of 5 cm focal length. The photo of the arrangement of the optical components and samples is shown in Figure 8 (on the right). The samples were raster-scanned by position-synchronized measurements in Cartesian coordinates [22]. A sensitive lock-in detection technique was used with modulation frequency and time constant being set to 1.46 kHz and 10 ms, respectively.

**Figure 9:**
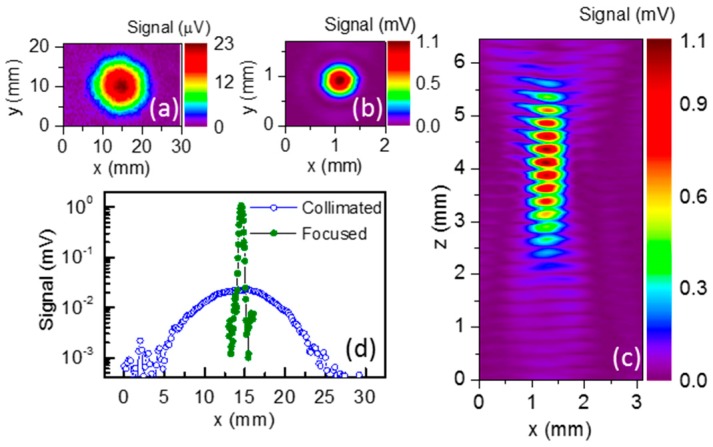
Shape of collimated (**a**) and focused with the TZP lens (**b**) THz beam of 584 GHz frequency. Note the difference in scales; (**c**) Beam shape along the optical axis after the diffractive lens; (**d**) Cross-section of collimated and focused THz radiation at peak intensity area. In all measurements the pixel size was 50 × 50 µm^2^, except (a)—200 × 500 µm^2^.

We have designed the TZP component to manipulate the Gaussian beam of 584 GHz frequency. The diffractive lens design was similar to the conventional Fresnel lens with the main difference being the integration of the resonant cross shape apertures inside open regions [30,31]. Resonant apertures of the length K = 260 µm, width M = 30 µm, and period L = 300 µm were used to obtain the peak of transmittance at the desired frequency [41]. The focal distance and diameter of the TZP were selected to be 10 mm and 16.5 mm, respectively. The diffractive lens was fabricated from 30 µm thick molybdenum foil by the direct laser writing. Typical performances of the developed diffractive component are shown in Figure 9. The NA of the TZP was measured by three-dimensional Gaussian beam profiling [39]. The NA value was estimated to be about 0.62.

**Figure 10:**
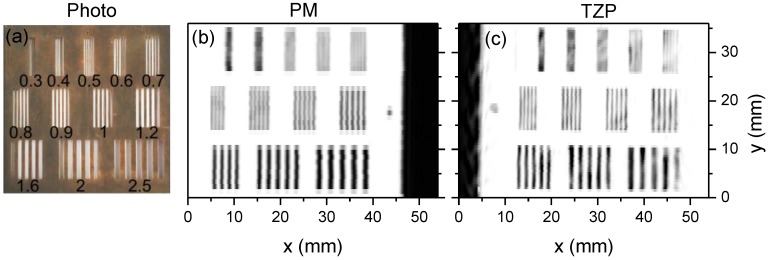
A photo of the resolution target consisting of a set of periodic metal stripes with the period indicated by number in mm (**a**). THz image of the resolution target at 584 GHz frequency obtained by using commercial PM (**b**) and novel diffractive lenses (**c**). Black color in the THz images corresponds to the maximum of transmittance.

**Figure 11:**
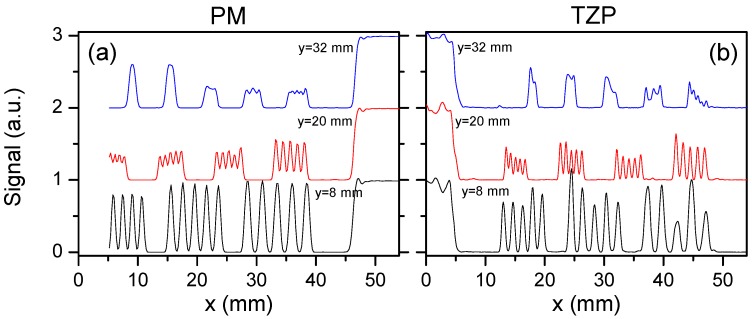
A cross section profile along the x-axis of the THz image at positions *y* = 8, 20, and 33 mm measured using different lenses: PM (**a**) and TZP (**b**).

The performance of the THz system based on the TZP lens *versus* a commercial off-axis PM was compared by imaging a spatial resolution target. The diameter and focal length of the PM were of 2 inches leading to the NA = 0.45. This was the highest NA that was found on the components market. Imaging results of the resolution target are shown in Figure 10 and Figure 11. It is seen that the system equipped with diffractive component TZP provided a much better spatial resolution. Periodic stripes were distinguishable if the period was not smaller than 0.6 mm in the case of TZP lens; note that the resolution was limited by the wavelength of used THz radiation. And in the case of off-axis PM, the smallest period of stripes was measured of about 0.8 mm. Thus, the imaging system with the TZP lens exibited improvement in spatial resolution of up to 25%.

## 5. A Compact THz Imaging System for Medical Applications

First dark-field THz imaging with the intention of enhancing image contrast through the analysis of scattering and diffraction signatures was demonstrated back in 2001 [33]. That time the samples contained obvious area of fat, skin with hairs, connective tissue and the tumor. Dark-field imaging discriminated between scattering/diffraction losses and absorption losses and demonstrated enhanced image contrast at boundaries and edges. The development of confocal microscopy THz systems deserved particular attention because with the confocal spatial filtering it was possible to improve the resolution and contrast of the image. Confocal THz systems based on powerful THz sources such as gas THz lasers and cryogenically cooled QCLs have been proposed [25,42]. As THz radiation may penetrate into the skin to a depth of 10 mm or deeper, the development of THz confocal microscopy systems for medicine is of prime interest. The THz-TDS spectroscopy results of a human basal cell carcinoma revealed that increased refractive index and absorption coefficient were the sources of contrast at THz frequencies and that these changes were consistent with an increase of water content in the tissue [37]. In addition, the THz TDS spectra of dehydrated and paraffin-embedded human colon tissues revealed that a higher percentage of water in cancerous tissues was not the only contributing factor to the contrast [35]. Higher refractive index and absorption coefficient were observed in neoplastic tissues by measuring spectra at several points in line along the sample. Such research reinforced the feasibility of THz techniques for early stage colon cancer detection and various THz imaging systems were developed for this purpose [38,43]. Contrast up to 23% between the neoplastic and non-neoplastic tissues was observed in THz absorption and reflection images averaged over the specified regions.

### 5.1. Demonstration of Compact THz Imaging System Performance

**Figure 12:**
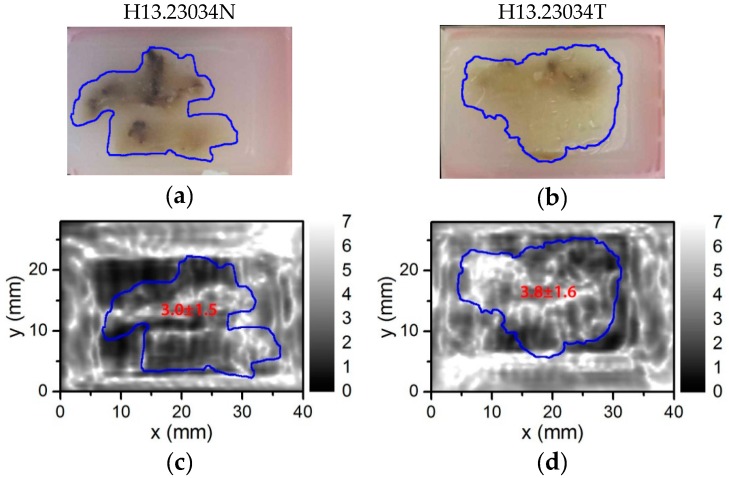
A digital photograph (**a**,**b**) and THz image (**c**,**d**) of the histopathologic sections of non-neoplastic and neoplastic colon tissue placed in a paraffin block. The control sample is presented on the left-hand side (**a**,**c**) and adenocarcinoma—on the right-hand side (**b**,**d**) column. The white color in the THz image corresponds to higher THz absorbance. The blue line indicates the contour of the tissue and the number—an averaged value of THz absorption inside the contour.

**Figure 13:**
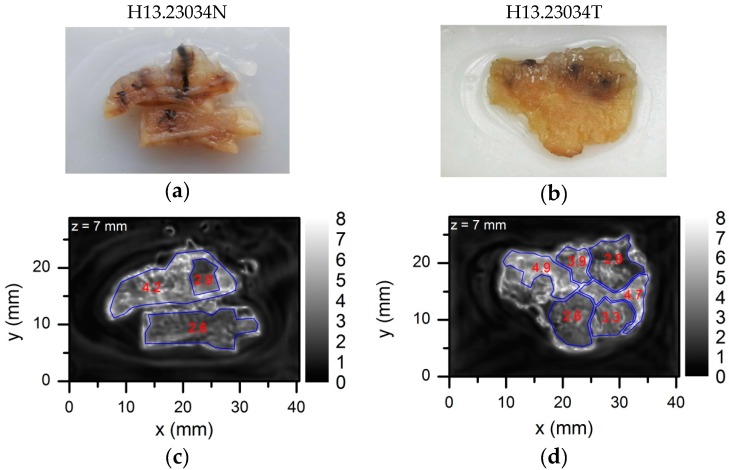
A digital photograph (**a**,**b**) and THz image (**c**,**d**) of the same histopathologic sections as in Figure 12 but the samples were taken out of paraffin and placed on a 2 mm thick PE plate and the THz imaging was performed employing the TZP lens. The control sample is on the left-hand side (**a**,**c**) and adenocarcinoma—on the right-hand side (**b**,**d**) column. The white color in the THz image corresponds to higher THz absorbance. Suspicious regions were indicated by a blue line. A number inside each region presents the averaged value of THz absorption.

In order to show suitability of a compact THz imaging system for medical applications, we used histological sections with colon cancer for the experiments [43,44]. Digital photos of the embedded in paraffin samples and free-laying ones are shown in the top line of Figure 12 and Figure 13, respectively. The samples were obtained of the patient (#H13.23034) by the right hemicolectomy procedure via selection of the specimens and further fixation in formalin and after that in paraffin blocks as described elsewhere [43,44]. The histologic analysis classified sample #H13.23034T as colon adenocarcinoma pT3 N0 Mx in the TNM system with the original tumor size of about 4 cm × 3.5 cm × 1.5 cm. The second sample #H13.23034N was obtained of the non-neoplastic tumor via the same surgery procedures and was studied for comparison purposes.

The samples were measured in transmission geometry, *i.e.* the radiation of THz frequency scattered and transmitted through the sample was collected with the L2 lens and detected with the NBMS detector D (see also Figure 8). In this experiment a commercial off-axis PM with the highest NA was used instead of the TZP lens. We note that the confocal spatial filtering was not applied.

The THz image of the paraffin embedded samples at 584 GHz frequency is shown in Figure 12. The mean value of the THz absorption was obtained via averaging measurements inside the range of interest (ROI) region indicated by a blue line contour. An averaged and standard deviation values were obtained for each ROI region and were shown in Figure 12. The adenocarcinoma regions can be observed as more THz absorbing regions even in dehydrated tissues due to increased radiation scattering caused by structural changes [43]. In this case, an averaged values of the THz absorption for samples H13.23034T and H13.23034N were 3.8 ± 1.6 and 3.0 ± 1.5, respectively. Systematic investigation allows drawing statistically significant assessments distinguishing non-neoplastic and neoplastic tissues [43]. Therefore one sample measurement even with high NA optics is not always sufficient. As shown in Figure 12, the tissue contour and different absorption regions in the THz image were blurred out and did not obviously correspond to the visible image most probably due to the interference from the paraffin blocks and surroundings of the sample holder.

**Figure 14:**
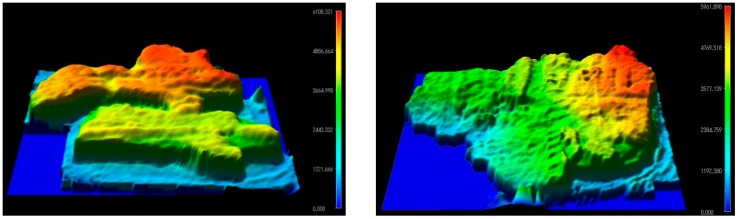
Thickness and surface profiles of sample H13.23034N (left-hand side) and H13.23034T (right-hand side) obtained with a three-dimensional digital microscope. Note the differences between the sample structure and THz absorption regions at a virtual cross section layer shown in Figure 13 and Supplementary Materials for the sample H13.23034N (Animation1.gif) and H13.23034T (Animation2.gif), respectively.

To overcome these limits, the paraffin embedded sample was placed on a 2 mm thick PE plate and heated up to temperature of 50 °C just enough to melt the paraffin away. A digital photo of the sample sticked to the PE plate via residual paraffin is shown in Figure 13. The measurements were performed with the THz imaging system taking preference for the TZP lens with NA = 0.62. The samples were oriented normal to the THz beam and face to the TZP lens. Measured THz images are shown in Figure 13. In this case the interference effect was removed as the PE plate behind the tissues had constant thickness that facilitated identification of physical boundaries between different tissue regions. Suspicious regions of increased THz absorption were selected and marked by a blue line. A mean value of the THz absorption was found via measurements averaging inside the ROI region. The mean values found are shown in Figure 13. The topographical thickness of the sample was measured with a three-dimensional digital microscope (Hirox Europe Ltd., Limonest, France). Results are shown in Figure 14. Obviously, the change in the THz absorption of the neoplastic tissue (H13.23034T) does not correlate with variation in the sample thickness and morphology differently from the case of the non-neoplastic sample (H13.23034N). Moreover, the THz image of non-neoplastic sample demonstrated separate regions where averaged THz absorption value changed from 2.6 ± 0.6 up to 4.9 ± 1.1. Note that the main indication of the adenocarcinoma tissues is increased absorption of THz radiation [43,44]. For proper medical diagnostics one needs to perform more systematic research with more samples also accounting the results of topographical measurements. However, that is beyond the scope of this paper.

### 5.2. Towards Confocal THz Microscopy

Exploring the performance of the THz imaging system such as high signal to noise ratio provided by the NBMS’s and high spatial resolution provided by the TZP, there was an interest to emulate functionality of the confocal THz microscope as complementary tool for pathology. The axial resolution of the THz system based on the TZP lens was estimated by the Rayleigh length criterion [39]. The distance, along which the focused beam waist increased by factor of √2, was found to be of about 1.4 mm (see Figure 9c). The samples on PE plate were imaged in transmission mode with only slight focusing adjustment by moving the sample in axial-steps of 1 mm. In such a way a set of THz images at different focal planes were obtained at 584 GHz. The results for the sample H13.23034N and H13.23034T were animated and presented in a section of Supplementary Materials Animation1.gif and Animation2.gif, respectively. We demonstrate a virtual cross sections, roughly 1 mm deep, within the sample due to varying the ratio between transmitted and scattered THz radiation by the sample. Fine tissue structure and especially regions with different THz contrast were clearly observed. In the next stage, a confocal spatial filtering will be implemented to provide high quality THz images with fine details and better contrast within the objects.

## 6. Conclusions

The spectral performance of the THz sensors based on air-bridged Ti-microbolometers coupled with a dipole antenna has been measured experimentally by FTIR spectroscopy at room temperature. These sensors were optimized for operation at a selected frequency of 300 GHz or 600 GHz and were used to develop an all-electronic THz imaging system suitable for plastic package inspection and spectroscopic-spatial analysis of materials. Next to it, a thin and lightweight focusing component called the TZP lens with the numerical aperture of 0.62 has been developed and used to enhance the performance of the THz imaging system. This was demonstrated by imaging carcinoma-affected biological tissues with close to wavelength limited spatial resolution. The proposed compact THz imaging systems prove the maturity of THz technology for medicine and security applications.

## Supplementary Materials

The following are available online at http://www.mdpi.com/1424-8220/16/4/432/s1, the confocal THz imaging data for the sample H13.23034N (Animation1.gif) and H13.23034T (Animation2.gif) measured at a frequency of 584 GHz.

## Abbreviations

The following abbreviations are used in this manuscript:

THz	Terahertz
RT	Room temperature
NEP	Noise-equivalent-power
FET	Fielf effect transistor
TeraFET	Terahertz antenna-coupled fielf effect transistor
QCL	Quantum cascade laser
PTFE	Polytetrafluoroethylene
PE	Polyethylene
FTIR	Fourier transform infrared
NBMS	Narrow band microbolometer terahertz sensor
THz TDS	Terahertz time domain spectrometer
TZP	Terahertz zone plate with integrated resonant filter apertures
CMOS	Complementary metal-oxide semiconductor
DA	Double dipole antenna
CDA	Cross dipole antenna
PM	Parabolic mirror
D^∗^	Relative detectivity
SNR	Signal to noise ratio
ROI	Range of interest
