# Supplementary figures and images for: Spectroscopic Terahertz Imaging at Room Temperature Employing Microbolometer Terahertz Sensors and Its Application to the Study of Carcinoma Tissues
Source: Sensors (Basel). 2016 Mar 25;16(4):432. doi: 10.3390/s16040432 (PMC4850946; doi:10.3390/s16040432)

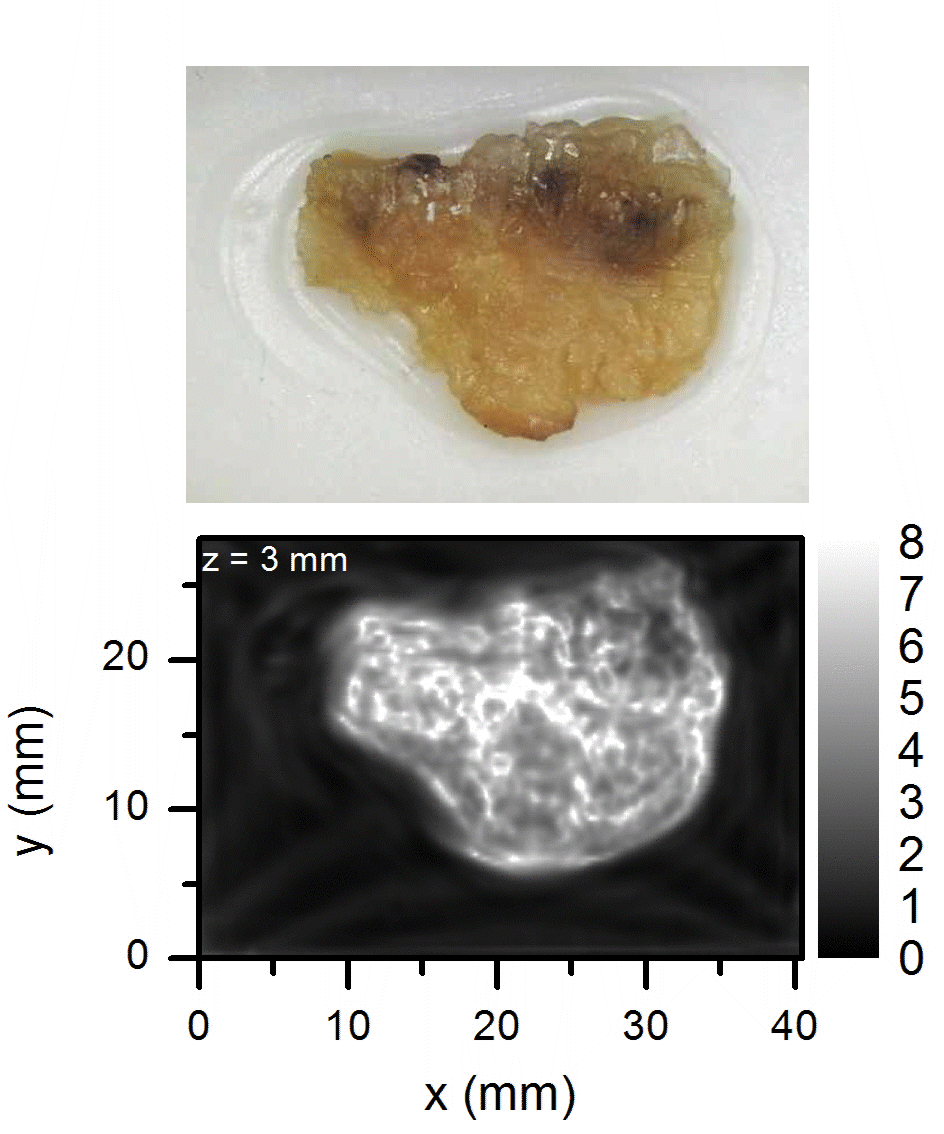

Supplement: Supplementary file 1 [file sensors-16-00432-s001.zip › Animation2_confocal THz imaging for the sample H13.23034T.gif]

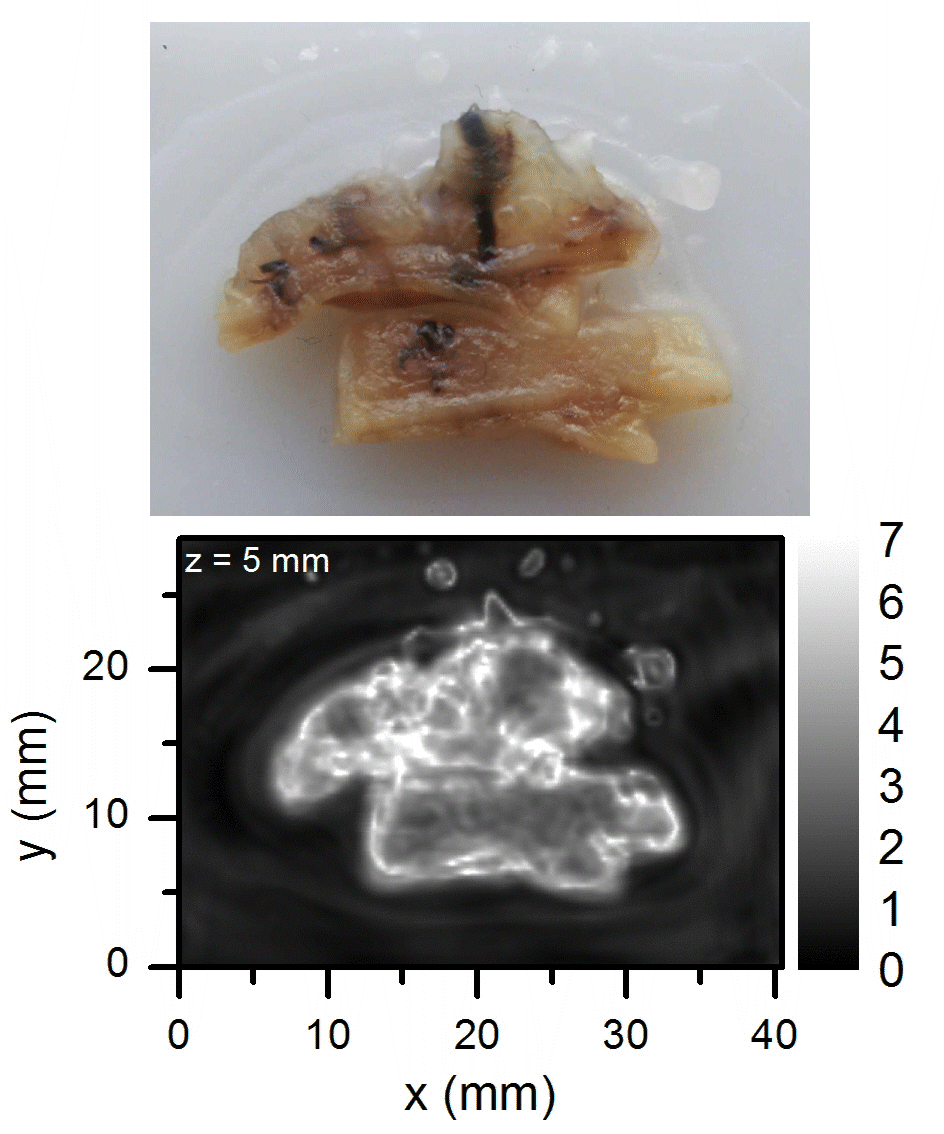

Supplement: Supplementary file 1 [file sensors-16-00432-s001.zip › Animation1_confocal THz imaging for the sample H13.23034N.gif]
